# Metabolic Changes and Antioxidant Response in *Ustilago maydis* Grown in Acetate

**DOI:** 10.3390/jof9070749

**Published:** 2023-07-14

**Authors:** Lucero Romero-Aguilar, Katia Daniela Hernández-Morfín, Guadalupe Guerra-Sánchez, Juan Pablo Pardo

**Affiliations:** 1 Departamento de Bioquímica, Facultad de Medicina, Universidad Nacional Autónoma de México, Circuito Interior, Ciudad Universitaria, Coyoacán, Ciudad de México C.P. 04510, Mexico; lusromaguila@bq.unam.mx; 2 Departamento de Microbiología, Escuela Nacional de Ciencias Biológicas, Instituto Politécnico Nacional, Carpio y Plan de Ayala S/N Santo Tomás, Miguel Hidalgo, Ciudad de México C.P. 11340, Mexico; hernandez_morfin1@hotmail.com

**Keywords:** acetate, oxidative stress, gluconeogenesis, glycolysis, lipid droplets, *Ustilago maydis*

## Abstract

*Ustilago maydis* is an important model to study intermediary and mitochondrial metabolism, among other processes. *U. maydis* can grow, at very different rates, on glucose, lactate, glycerol, and ethanol as carbon sources. Under nitrogen starvation and glucose as the only carbon source, this fungus synthesizes and accumulates neutral lipids in the form of lipid droplets (LD). In this work, we studied the accumulation of triacylglycerols in cells cultured in a medium containing acetate, a direct precursor of the acetyl-CoA required for the synthesis of fatty acids. The metabolic adaptation of cells to acetate was studied by measuring the activities of key enzymes involved in glycolysis, gluconeogenesis, and the pentose phosphate pathways. Since growth on acetate induces oxidative stress, the activities of some antioxidant enzymes were also assayed. The results show that cells grown in acetate plus nitrate did not increase the amount of LD, but increased the activities of glutathione reductase, glutathione peroxidase, catalase, and superoxide dismutase, suggesting a higher production of reactive oxygen species in cells growing in acetate. The phosphofructokinase-1 (PFK1) was the enzyme with the lowest specific activity in the glycolytic pathway, suggesting that PFK1 controls the flux of glycolysis. As expected, the activity of the phosphoenolpyruvate carboxykinase, a gluconeogenic enzyme, was present only in the acetate condition. In summary, in the presence of acetate as the only carbon source, *U. maydis* synthesized fatty acids, which were directed into the production of phospholipids and neutral lipids for biomass generation, but without any excessive accumulation of LD.

## 1. Introduction

*Ustilago maydis* is a biotrophic basidiomycete that infects maize (*Zea mays*). This microorganism is among the 10 most important models for the study of processes such as pathogenesis [1,2] signal transduction pathways involved in plant invasion and dimorphic transition [3,4], microtubule-dependent transport of mRNA [5], and the response to different types of stress [6,7,8,9]. Recently, *U. maydis* has been introduced as a good biotechnological model for the production of sesquiterpenes, glycolipids, itaconic acid, lipids, lipases [10,11], and the expression of heterologous proteins [12,13].

*U. maydis* is a fully aerobic fungus that grows on glucose as the preferred carbon source but can also use other carbon sources such as lactate, glycerol, and ethanol [14]. Glucose enters the glycolytic pathway and produces two molecules of pyruvate, which are oxidized to acetyl-CoA in the mitochondrial matrix. Acetyl-CoA is a key metabolic intermediate that serves as a precursor for the biosynthesis of various important chemical compounds, such as lipids and isoprenoids, the latter used as flavor and pigments in the pharmaceutical and food industry [15]. We previously showed that *U. maydis* produces a large amount of neutral lipids that are stored in a few large LD when it grows in a medium containing glucose as a carbon source but without a nitrogen source [16]. In addition, for the synthesis of the neutral lipids, the activities of glucose-6-phosphate dehydrogenase (G6PDH), 6-phosphogluconate dehydrogenase (6PGDH), and the cytosolic isocitrate dehydrogenase (NADP^+^-IDH) were important for NADPH generation [16]. Because acetate is the direct precursor for acetyl-CoA synthesis [17,18], we hypothesized that in this condition the synthesis of lipids would be favored. Therefore, in this work, we studied the production of neutral lipids as LD, quantified the triacylglycerol content, and determined the enzyme activities related to the lipid synthesis, carbon metabolism, and oxidative stress in *U. maydis* cells growing in acetate as the only carbon source and in the presence of nitrate as a nitrogen source. The results show that LD was not accumulated in cells cultured in acetate with nitrogen. Although the growth of *U. maydis* on acetate relied fully on the gluconeogenic pathway, the three regulatory glycolytic enzymes, hexokinase (HK), phosphofructokinase-1 (PFK1), and pyruvate kinase (PK) were active in this condition. The phosphoenolpyruvate carboxykinase (PEPCK) was present only in the acetate condition. Since some reports indicate that growth in acetate induces oxidative stress [6,19], we studied the production of hydrogen peroxide (H_2_O_2_) and determined the activities of the antioxidant enzymes glutathione reductase (GSH-Rd), glutathione peroxidase (GSH-Px), catalase (Cat), and superoxide dismutase (SOD). The goal of this work was to study the synthesis of neutral lipids using acetate as the precursor of acetyl-CoA and the metabolic adaptation of *U. maydis* to this carbon source.

## 2. Materials and Methods

### 2.1. Strains and Growth Conditions

*Ustilago maydis* wild-type FB2 (a2b2) was maintained in 25% glycerol (*v*/*v*) at −70 °C and recovered in Yeast Peptone Dextrose Agar (YPD-agar) (0.5% yeast extract, 0.25% bactopeptone, 0.5% glucose, and 2% agar). Cells were cultured in 50 mL of YPD medium for 24 h at 180 rpm and 28 °C, recovered by centrifugation at 3000× *g*, washed once with 50 mL of sterile distilled water, and suspended in sterile distilled water (1 mL H_2_O per g wet weight, pre-cultured cells). The pre-cultured cells were used to inoculate 60 U/L (final optical density at 600 nm of 0.06) of minimal medium with glucose or acetate, as a carbon source, then they were incubated at 28 °C, 200 rpm for different times. The minimal medium contained 1% glucose or 1% potassium acetate as a carbon source, 0.3% potassium nitrate as a nitrogen source, and 6.5 mL of a salt solution. The salt solution contains, per liter, 16 g KH_2_PO_4_, 4 g Na_2_SO_4_, 2 g KCl, 1 g CaCl_2_, and 8 mL of the trace elements solution (0.006% H_3_BO_3_, 0.014% MnCl_2_·4H_2_O, 0.04% ZnCl_2_, 0.004% Na_2_MoO_4_·2H_2_O, and 0.01% FeCl_3_·6H_2_O). The minimal medium was prepared according to [20].

### 2.2. Dry Weight Determination

Aliquots of 1.4–2.8 mL of cell cultures were collected at 24, 48, and 72 h and centrifuged in pre-weighed tubes at 16,000× *g* to eliminate the culture medium. Then, the tubes were placed in an oven at 70 °C. After 72 h, the weight of the empty tubes was subtracted from the weight of the tubes containing the dry sample.

### 2.3. Colony Forming Units

Aliquots from the culture media were collected and adjusted to an optical density at 600 nm (OD_600nm_) of 3 × 10^−4^. From this suspension, 10 µL were spread on YPD-agar and incubated at 28 °C for 48 h. Colony Forming Units (CFU) were counted, and the data were normalized to one milliliter considering the dilutions.

### 2.4. Total Number of Cells

The total number of cells was obtained with the Countess Automated Cell Counter II from Thermo Fisher Scientific, Waltham, MA, USA. Data were normalized to one milliliter considering the dilutions.

### 2.5. Glucose Consumption

Aliquots of 1 mL were withdrawn at the indicated times and centrifuged for 1 min at 16,000× *g*. The supernatant was recovered and used for the determination of glucose using a colorimetric kit based on the activity of glucose oxidase (Glucose-TR, Spinreact, Girona, Spain).

### 2.6. Flow Cytometry

Cells were fixed with 4% formaldehyde (15 min at room temperature) and washed twice with a 0.9% NaCl solution. For acquisition, cells were suspended at a final concentration of 5 × 10^6^ cells/mL, heavily vortexed, and briefly sonicated in a compact high-performance ultrasonic cleaning system (Fisher Scientific FS3). Per condition, 20,000 events were acquired with the flow cytometer MACSQUANT analyzer 10 (Miltenyi Biotec, Bergisch Gladbach, Germany) [21]. Four samples from independent cultures were analyzed.

### 2.7. Hydrogen Peroxide Production

Cells were cultured as mentioned. At 24, 48, and 72 h of incubation aliquots were withdrawn, washed once with sterile distilled water, and adjusted to an OD = 0.5 with distilled water. Production of H_2_O_2_ was followed by mixing 50 µL of the cell suspension with 50 µL of the Amplex™ Red Kit from Thermo Fisher Scientific [9].

### 2.8. Confocal Microscopy

Cells incubated in the presence of glucose or acetate with or without nitrogen source were harvested, washed once with distilled water, fixed with 4% formaldehyde (15 min at room temperature), and washed twice with 0.9% NaCl solution. Then, the pellet was resuspended to an OD_600nm_ of 5, and cells were stained with 5 µM of BODIPY^®^ suspended in 0.5 M of KI. Cells were mounted on Silane-Prep Slides (Sigma-Aldrich, St. Louis, MO, USA) and imaged on a confocal microscope (Zeiss LSM5 Pascal, Carl Zeiss GmbH, Göttingen, Germany) with an oil-immersion 100X N.A. 1.3 objective. The images were analyzed with Fiji software (https://fiji.sc/).

### 2.9. Triacylglycerol Quantification

Lipid extraction was performed according to Jouihan et al., 2012 [22]. Cells were harvested and 500 mg of wet weight were resuspended in 500 µL of 30% (*p*/*v*) KOH: ethanol (1:2 *v*/*v*) and incubated at 60 °C overnight. The sample was mixed with 500 µL of ethanol: H_2_O (1:1 *v*/*v*) and centrifuged at 22,000× *g* for 5 min. Approximately 1 mL of the supernatant was recovered and 200 µL of ethanol: H_2_O solution (1:1 *v*/*v*) was added. The sample was gently vortexed and 200 µL was withdrawn and added to a tube containing 215 µL of 1 M MgCl_2_ precooled at 4 °C, and the sample was incubated for 10 min at 4 °C. Centrifugation was repeated. The supernatant was recovered and used to analyze the triacylglycerol content using the SPINREACT Triglycerides-LQ kit. Data were expressed as a function of the grams of dry weight (gDW).

### 2.10. Glycogen Quantification

The determination was carried out according to Zhang 2012 [23]. Cells were treated and harvested as described. A sample of 250 mg of wet weight was hydrolyzed with 0.5 mL of 2 M HCl in a boiling water bath for 1 h with vigorous shaking every 10 min. In parallel to the acid hydrolysis (HCl), alkaline hydrolysis was performed, and 2 M NaOH replaced the HCl. The pH of the hydrolysis products was neutralized to 6–8 with NaOH or HCl, respectively. The samples were then cooled at room temperature and centrifuged at 22,000× *g* for 10 min at 4 °C. The supernatant was used to determine the glucose with the kit SPINREACT-glucose-LQ.

### 2.11. Preparation of Cell-Free Extracts

Cells were harvested and resuspended with the following lysis buffer: 50 mM KH_2_PO_4_, 30 mM HEPES, 5 mM EDTA, 20% glycerol, 1 mM PMSF, pH 7.0, and mixed with a volume of glass beads (0.5 mm). Cells were subjected to five pulses of 1 min at 4800 oscillations/min using the mini bead-beater equipment with intervals of 1 min incubation in an ice water bath. The lysate was clarified by centrifugation at 20,000× *g* in a SCILOGEX SCI24R high-speed refrigerated microcentrifuge. Protein was determined by the Lowry assay [24].

### 2.12. Glutathione Reductase

The activity was determined by following the oxidation of NADPH at 340 nm. The assay contained 20 mM phosphate buffer (NaH_2_PO_4_/Na_2_HPO_4_), 1 mM EDTA (Buffer A), 0.15 mM NADPH, 1 mM oxidized glutathione, pH 7.0. The reaction was started by adding the cell-free extract. The specific activity was calculated using an extinction coefficient of 6.22 mM^−1^ cm^−1^ for NADPH and considering the protein in the assay [25,26].

### 2.13. Glutathione Peroxidase

The activity was assayed by following the oxidation of NADPH at 340 nm. The assay had Buffer A, 0.15 mM NADPH, 1 mM reduced glutathione, 0.1 mM cumene hydroperoxide, pH 7.0. The reaction was started by adding the cell-free extract. The specific activity was calculated using an extinction coefficient of 6.22 mM^−1^ cm^−1^ for NADPH and the protein in the assay [27,28].

### 2.14. Superoxide Dismutase

The activity was determined as the percentage of inhibition of nitroblue tetrazolium (NBT) reduction. The reduction of NBT was followed at 560 nm. The buffer assay contained Buffer A, 0.2 mM NBT, 0.6% Triton^TM^-X 100, 50 mM sodium carbonate (Na_2_CO_3_), and 20 mM hydroxylamine hydrochloride (NH_2_OH.HCl), pH 7.0. The mixture was incubated for ten minutes at 37 °C. Then, an aliquot of the cell-free extract (20 μL) was added, and readings were recorded for 7 min. The percentage of inhibition was calculated based on the initial slope given by the reduction of NBT [29,30].

### 2.15. Catalase-Peroxidase

The activity was determined by following the disappearance of H_2_O_2_ at 240 nm. The reaction mixture contained 20 mM phosphate buffer (NaH_2_PO_4_/Na_2_HPO_4_), pH 7.0, and 30 mM H_2_O_2_. The reaction was started with the addition of an aliquot of 10 μL of the cell-free extract [31].

### 2.16. Hexokinase

The activity was assayed in the buffer containing 30 mM HEPES pH 7.3, 3 mM MgCl_2_, 100 mM KCl, 10 mM KH_2_PO_4_ (Buffer B), 200 mM sorbitol, 1 mM NAD^+^, 5 mM glucose, 3 mM Mg-ATP, 0.8 U glucose-6-phosphate dehydrogenase. The reaction was started with the addition of the cell-free extract. The specific activity was calculated using an extinction coefficient of 6.22 mM^−1^ cm^−1^ for NADH and the protein in the assay [20].

### 2.17. Phosphofructokinase

The reaction mixture contained Buffer B, 3 mM Mg-ATP, 0.15 mM NADH, 2.5 mM fructose-6-phosphate, 0.2 U lactate dehydrogenase (LDH), 1 U pyruvate kinase (PK), 100 mM sorbitol, and cell-free extract. The specific activity was calculated using an extinction coefficient of 6.22 mM^−1^ cm^−1^ for NADH and the protein in the assay [20].

### 2.18. Pyruvate Kinase

The reaction mixture was composed of Buffer B, 3 mM Mg-ADP, 0.15 mM de NADH, 1 mM phosphoenolpyruvate (PEP), 0.4 U LDH, and cell-free extract. The specific activity was calculated using an extinction coefficient of 6.22 mM^−1^ cm^−1^ for NADH and the protein in the assay [20].

### 2.19. Glucose-6-Phosphate Dehydrogenase

The activity was assayed in a buffer containing Buffer B, 2 mM glucose-6-phosphate, 0.6 mM NADP^+^, and cell-free extract. The specific activity was calculated using an extinction coefficient of 6.22 mM^−1^ cm^−1^ for NADPH and the protein in the assay [32].

### 2.20. Fructose-1, 6-Bisphosphatase

The activity was assayed in Buffer B, 1mM NAD^+^, 0.75 U phosphoglucoisomerase (PGI), and 0.8 U G6PDH and cell-free extract. The specific activity was calculated using an extinction coefficient of 6.22 mM^−1^ cm^−1^ for NADH and the protein in the assay [33].

### 2.21. Pyruvate Carboxylase

The activity was assayed in Buffer B, 9 mM pyruvic acid, 0.3 mM acetyl-CoA, 1 U malate dehydrogenase, 3 mM Mg-ATP, 0.15 mM NADH, 15 mM NaHCO_3_, and cell-free extract. The specific activity was calculated using an extinction coefficient of 6.22 mM^−1^ cm^−1^ for NADH and the protein in the assay [34].

### 2.22. Fatty Acid Synthase

The activity was assayed according to Kelly et al. (1986) [35]. The reaction mixture contained Buffer B, 0.4 mM EDTA, 200 μM NADPH, 66 μM Acetyl-CoA, 200 μM malonyl-CoA, and cell-free extract. The specific activity was calculated using an extinction coefficient of 6.22 mM^−1^ cm^−1^ for NADH and the protein in the assay.

### 2.23. Phosphoenolpyruvate Carboxykinase

The activity was assayed according to Lee et al., 1981 [36]. The buffer of the reaction contained: 45 mM NaHCO_3_, 2 mM phosphoenolpyruvate, 3 mM MnCl_2_, 0.15 mM NADH, 0.75 U malate dehydrogenase, 3 mM ADP, and cell-free extract. The specific activity was calculated using the extinction coefficient of 6.22 mM^−1^ cm^−1^ for NADH.

### 2.24. pH Determination

An aliquot of 1.5 mL of the culture medium was withdrawn at the indicated times and centrifuged at 18,600× *g* for 5 min in a tabletop centrifuge. The pH of the supernatants was measured with a glass electrode connected to a pH meter.

### 2.25. Cell Viability by Flow Cytometry

From a 72 h cell culture, an aliquot of 2 × 10^6^ cells/mL was harvested by centrifugation at 14,000 rpm in a top table centrifuge, washed once with a buffer containing 137 mM NaCl, 2.7 mM KCl, 10 mM Na_2_HPO_4_, 1.8 mM KH_2_PO4, pH 7.4 (PBS), and resuspended with a solution of PBS containing the 6X Ghost Dye™ Violet 450 [PBS-GD450]. Then, the samples were incubated in the dark at 27 °C for 50 min without shaking. Cells were recovered by centrifugation, washed once with PBS, and resuspended in fresh PBS. For a positive control (cells with compromised plasma membrane), cells were incubated in a water bath at 85 °C for 5 min, and then the sample was centrifuged and treated with the PBS-GD450 solution as previously described. Acquisition in the cytometer was conducted as mentioned in 2.6. Per condition, 20,000 events were acquired with the flow cytometer MACSQUANT analyzer 10 (Miltenyi Biotec, Germany).

## 3. Results

### 3.1. Growth of Ustilago Maydis in Media with Acetate or Glucose

**Figure 1:**
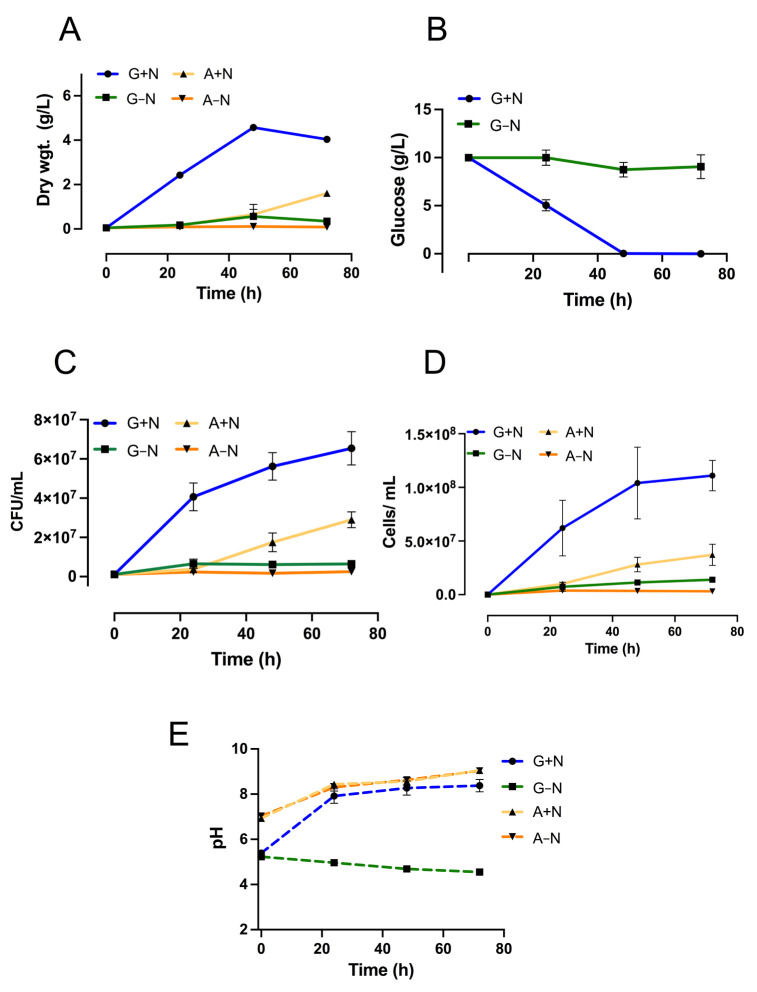
Effect of carbon sources on the vegetative growth and cell viability of *U. maydis*. Cells were grown in minimal media containing 1% acetate with (A+N) or without nitrogen (A-N) or minimal media containing 1% glucose with (G+N) or without nitrogen (G-N). Aliquots were withdrawn at the indicated times. (**A**) gDW/L. (**B**) glucose consumption (g/L). (**C**) colony-forming units (CFU/mL). (**D**) Cell count (Cells/mL). (**E**) pH values. Data are shown as the mean and standard deviation of 4 independent experiments.

**Figure 2:**
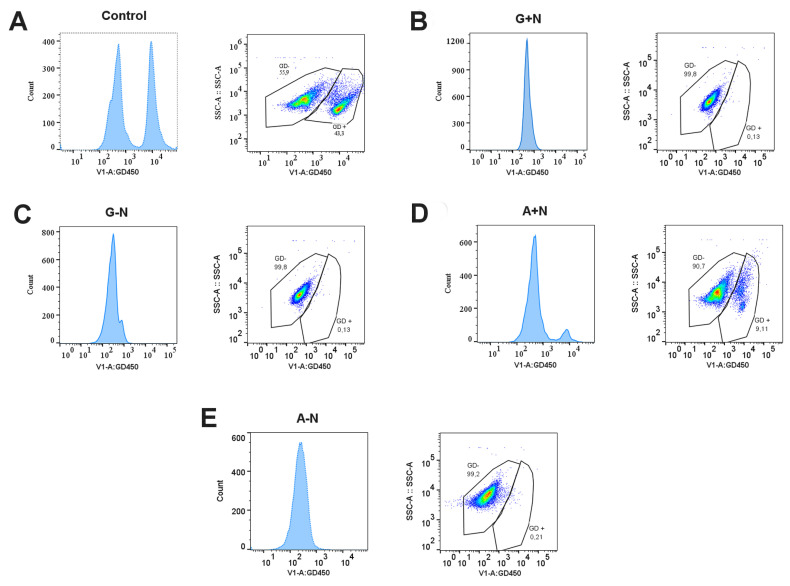
Cell viability by flow cytometry. Cells were grown in minimal media with acetate or glucose as a carbon source, stained with GD450, and analyzed by flow cytometry. (**A**) Control cells; cells were killed by a heat shock. The histogram in the left represents viable cells (negative) and at the right dead cells (positive). In the scatter plot, positive and negative cells are gating and the percentage of each population is indicated. (**B**) Cells cultivated in minimal medium with glucose plus nitrogen, G+N. (**C**) Glucose without nitrogen, G-N. (**D**,**E**) Acetate with or without a nitrogen source, respectively. Representative graphs from 4 independent experiments are shown.

During cell growth, nitrogen is important for the synthesis of proteins and nucleic acids, while carbon is required for the synthesis of practically all the organic molecules incorporated into the biomass. In general, microorganisms can grow in different carbon sources although at different rates and yields. Therefore, we examined the capacity of *U. maydis* to grow in a minimal medium with acetate or glucose as a carbon source and in the presence (A+N, G+N) or absence (A-N, G-N) of nitrate as a nitrogen source. Figure 1A shows that in the presence of nitrate, biomass production was four times higher in glucose than in acetate. There was a time-dependent increase of DW during the first 48 h of culture in G+N, but biomass was smaller at 72 h (Figure 1A). This unexpected behavior is probably because cells consumed the glucose in the first 48 h of culture (Figure 1B). However, in the first 48 h of culture, the increase in biomass was closely related to glucose consumption (Figure 1B), the number of viable cells (CFU/mL) (Figure 1C), and the total number of cells/mL (Figure 1D). However, despite the decrease in DW at 72 h of culture in G+N, the total number of cells, the CFU/mL and cell viability remained relatively constant (Figure 1C,D and Figure 2B). Cell growth in G+N was associated with an increase in the pH of the medium, but in G-N there was acidification of the medium (Figure 1E). In contrast, in the presence of acetate, there was an alkalinization, regardless of the presence of nitrate (Figure 1E). Regarding the growth in A+N, biomass generation increased from 0.65 gDW at 48 h (14.3% of G+N) to 1.60 gDW at 72 h (39.8% of G+N) (Figure 1A). Both viable cells (Figure 1C) and the total number of cells (Figure 1D) increased with the culture time. It is worth to mention that the viability of cells grown for 72 h in the A+N medium, was above 80% (Figure 2D). In agreement with a previous report [16], in the absence of a nitrogen source cells stopped growing, and biomass production was low, reaching a value of 0.34 g/L in G-N (8.6% of G+N) and 0.08 g/L in A-N (3.4% of G+N) (Figure 1A) at 72 h of culture. The consumption of glucose by cells was minimal in the absence of nitrate (approximately 1g), but enough to explain the production of biomass and LD. In contrast to some reports [6], the viability of *U. maydis* cultured for 72 h in A+N was not affected (Figure 2D). Cell viability determined by flow cytometry in the A+N condition was 84 ± 4.5 while in the A-N condition was 95 ± 0.1. It is interesting that even after 72 h without a nitrogen source, cells are still viable (Figure 2D,E). The results indicate that *U. maydis* can grow without a loss of viability in a medium containing acetate as the only carbon source (Figure 2D,E).

### 3.2. Acetate Does Not Promote Lipid Accumulation

**Figure 3:**
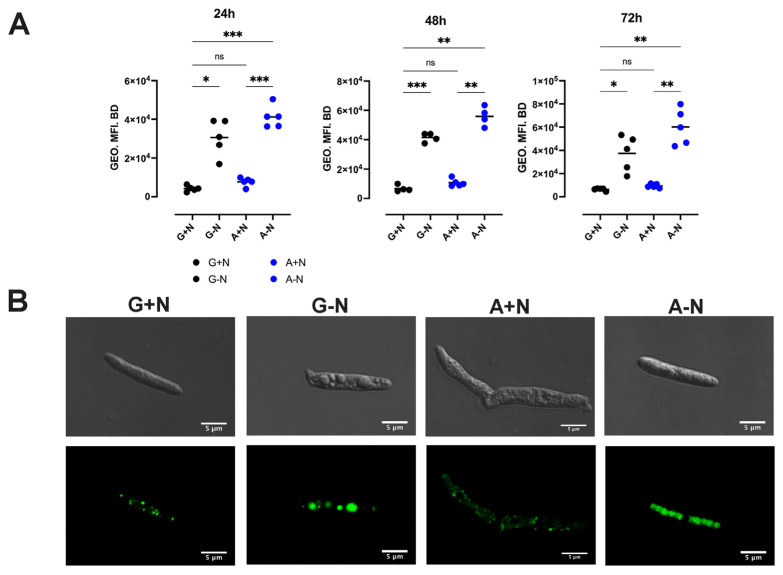
Lipid accumulation analyzed by fluorescence. Lipid droplet content was evaluated by the geometric mean of fluorescence intensity of BODIPY (GEO.MFI.BD). (**A**) GEO.MFI.BD. (**B**) Confocal microscopy of LD filled with TAGs in cells grown for 48 h in minimal media of different compositions. Data are shown as the mean and standard deviation of 4 independent experiments. White bar = 5 µm. Significant difference at * *p* < 0.05, ** *p* <0.005 *** *p* < 0.0005 comparing G+N with the other conditions A+N, A-N, and G-N (ANOVA).

*U. maydis* accumulates high amounts of triacylglycerols in intracellular LD when cultured in G-N [16]. Since acetate is one of the substrates of the acetyl-CoA synthetase (ACS), the enzyme that produces acetyl-CoA from acetate, which in turn is the substrate for the lipid synthesis [37,38], we wondered if the metabolism of acetate in *U. maydis* was associated with the accumulation of LD. Will it produce an increase in acetyl-CoA synthesis and, in consequence, an accumulation of lipids? Therefore, we studied the content of triacylglycerols (TAGs) and LD in *U. maydis*. Cells were cultured in acetate or glucose in the presence or absence of nitrate, and at 24 h and 48 h of incubation, aliquots were withdrawn to determine the content of TAGs by an enzymatic assay and the formation of LD by flow cytometry and confocal microscopy using BODIPY^®^ (Figure 3A,B). The low amount of biomass obtained in the condition of A-N made it impossible to determine TAGs, glycogen, and enzyme activities in cell-free extracts.

**Figure 4:**
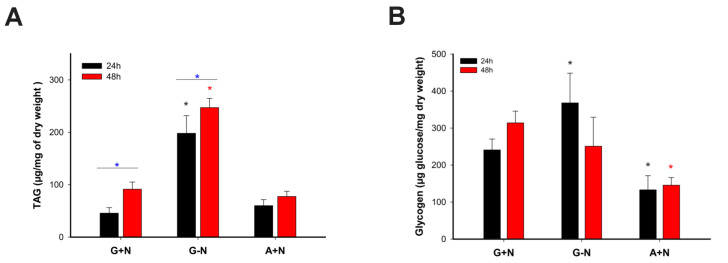
Triacylglycerols and glycogen content. Triacylglycerols and glycogen were determined by an enzyme-linked assay. (**A**) Triacylglycerols and (**B**) Glycogen content. Growth conditions are indicated in the figure. Data are shown as the mean and standard deviation of 4 independent experiments. Significant difference between the samples were calculated at *p* < 0.05 using the ANOVA test. Blue asterisk: G+N 24 h vs. G+N 48 h, G-N 24 h, and G-N 48 h. Dark asterisk: G+N 24 h vs. G-N 24 h. Red asterisk: G+N 48 h vs. G-N 48 h.

As can be seen in Figure 3A, the content of TAGs in *U. maydis* was similar in G+N and A+N; the geometric mean fluorescence intensity (GEO.MFI.BD) was 6576 ± 1092 and 10,628 ± 1142, respectively. In G-N and A-N, the content of TAGs was 2.5 to 4 times higher than in media containing carbon and nitrogen sources. At 48 h of culture (Figure 3A), the GEO.MFI.BD was 45,398 ± 9920 and 55,985 ± 3281 for G-N and A-N, respectively. Against our initial hypothesis, cells growing in A+N did not accumulate LD. However, the accumulation of LD by cells cultured in A-N was similar to that previously reported for G-N (Figure 3A,B). Consistent with this result, confocal microscopy showed that LD content in *U. maydis* was higher in G-N and A-N (Figure 3B). Quantification of TAGs corroborated the accumulation of TAGs in LD. In A+N and G+N, the content of TAGs was around 100 µg TAG/mgDW, while in G-N increased to 250 µg TAG/mgDW (Figure 4A). The results indicate that *U. maydis* cells growing in acetate as the only carbon source can synthesize the molecules needed for biomass generation, including TAGs and phospholipids, but in the presence of a nitrogen source, there was not a significant accumulation of TAGs in the form of LD (Figure 3A,B).

### 3.3. Acetate Promotes Glycogen Synthesis

Cell growth on acetate depends on gluconeogenesis for the synthesis of glucose, which, in turn, can be stored as glycogen. So, the content of glycogen in cells cultured in different culture media was quantified (Figure 4B). In all conditions assayed there was an accumulation of glycogen in cells, being the A+N condition the one in which cells accumulated the smallest amount of glycogen, 150 µg/mgDW. Interestingly, G-N was the condition with the major accumulation of glycogen at 24 h, 350 µg/mgDW (Figure 4B). In G+N, glycogen accumulation at 24 h and 48 h was 241 and 315 µg/mgDW, respectively (Figure 4B). The results indicate that cells synthesize glycogen from glucose and to a lesser extent from acetate.

### 3.4. Acetate Carbons Are Used to Activate Gluconeogenesis

**Figure 5:**
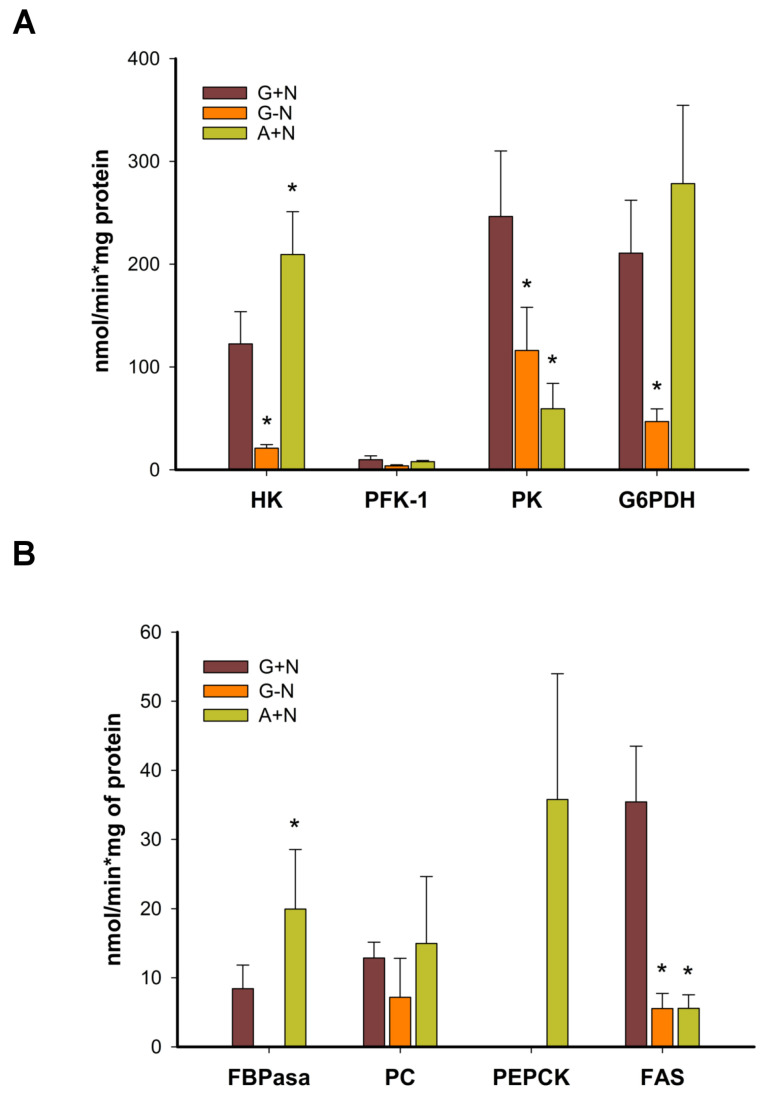
Specific activities of selected cytosolic enzymes. (**A**) glycolytic enzymes and the pentose phosphate pathway. (**B**) gluconeogenic enzymes and the FAS were determined in a clarified extract obtained from cells cultured for 24 h. Data are shown as the mean and standard deviation of 4 independent experiments. * Indicates a significant difference at *p* < 0.05, comparing minimal media with glucose and nitrate with the other conditions (ANOVA). Abbreviations: minimal media with glucose and nitrogen (G+N), glucose without nitrogen (G-N), acetate plus nitrogen (A+N), and acetate without nitrogen (A-N). HK, hexokinase; PFK-1, phosphofructokinase 1; PK, pyruvate kinase; G6PDH, glucose-6-phosphate dehydrogenase; FBPase, fructose 1,6-bisphosphatase; PC, pyruvate carboxylase; PEPCK, phosphoenolpyruvate carboxykinase; FAS, fatty acid synthase.

Because biomass production and glycogen accumulation in A+N indicate the activation of gluconeogenesis, we quantified the specific activity of the key enzymes involved in this metabolic pathway. Furthermore, the presence of glycogen and TAGs under this condition led us to study the activity of some of the enzymes involved in glycolysis and the pentose phosphate pathway (Figure 5). HK is essential for the assimilation of glucose obtained from glycogen degradation (in addition to glucose-1-phosphate), and the pentose phosphate pathway is important for the production of NADPH, an important factor in the synthesis of fatty acids. Clarified extracts of cells grown in the different experimental conditions were used to assay the activity of the enzymes. Specific activities are reported as nmol min^−1^ mg protein^−1^. Interestingly, the HK, PFK1, and PK were active under all conditions. The HK showed the highest activity in A+N (210 ± 41) compared to the G+N (122 ± 31) (Figure 5A). The lowest activity was found in G-N, 21 ± 3.5. PK had the following activities: 246 ± 64 in G+N, 59 ± 24 in A+N, and 116 ± 42 in G-N (Figure 5A). The PFK1 had the smallest activity in all conditions, indicating that PFK1 is the enzyme that has most of the control of the glycolytic flux. Its activity was 7.9 ± 1.16 in A+N, 9.8 ± 4 in G+N, and 3.8 ± 1.0 in G-N. In all cases, the activities of HK and PK were higher than the activity of PFK1 (Figure 5A, Table 1).

As mentioned, NADPH is needed for the synthesis of important biomolecules such as deoxyribonucleotides and fatty acids, but NADPH is also essential for the response of cells against oxidative stress. Since G6PDH is involved in the production of NADPH and its reaction is directly connected to glucose 6-phosphate in either gluconeogenesis or glycolysis, we measured its activity in cell-free extracts. Cells growing in A+N had a specific activity of 278 ± 76, 210 ± 51 for cells in G+N, and 47 ± 12 in the G-N condition (Figure 5A, Table 1).

Regarding gluconeogenic enzymes, PEPCK activity was not detected in G+N or G-N, but it was found in A+N with an activity of 36 ± 18 (Figure 5B, Table 1). In contrast, the PC was active in all conditions, with similar specific activities (Figure 5B). The activity of fructose-6-bisphosphatase (FBPase) was found in cells growing in G+N and A+N, but not in G-N (Figure 5B). In A+N, the enzyme had a specific activity of 20 ± 8.6, and in G+N 8.4 ± 3.4 nmol min^−1^ mg protein^−1^ (Figure 5B).

Interestingly, the activity of the fatty acid synthase (FAS) was practically the same (around 5.5 ± 2) in A+N and G-N, but in both conditions, the activity was smaller than that obtained in G+N (35 ± 8) (Figure 5B). Taken together, the results show that with acetate as the only carbon source, the three key glycolytic enzymes are present in cells with specific activities similar to those obtained in the presence of glucose. The PEPCK was expressed only in the gluconeogenic condition given by A+N, and the G6PDH and the FAS were expressed in all conditions. Specific activities are summarized in Table 1.

### 3.5. Acetate Induces the Production of Hydrogen Peroxide

**Figure 6:**
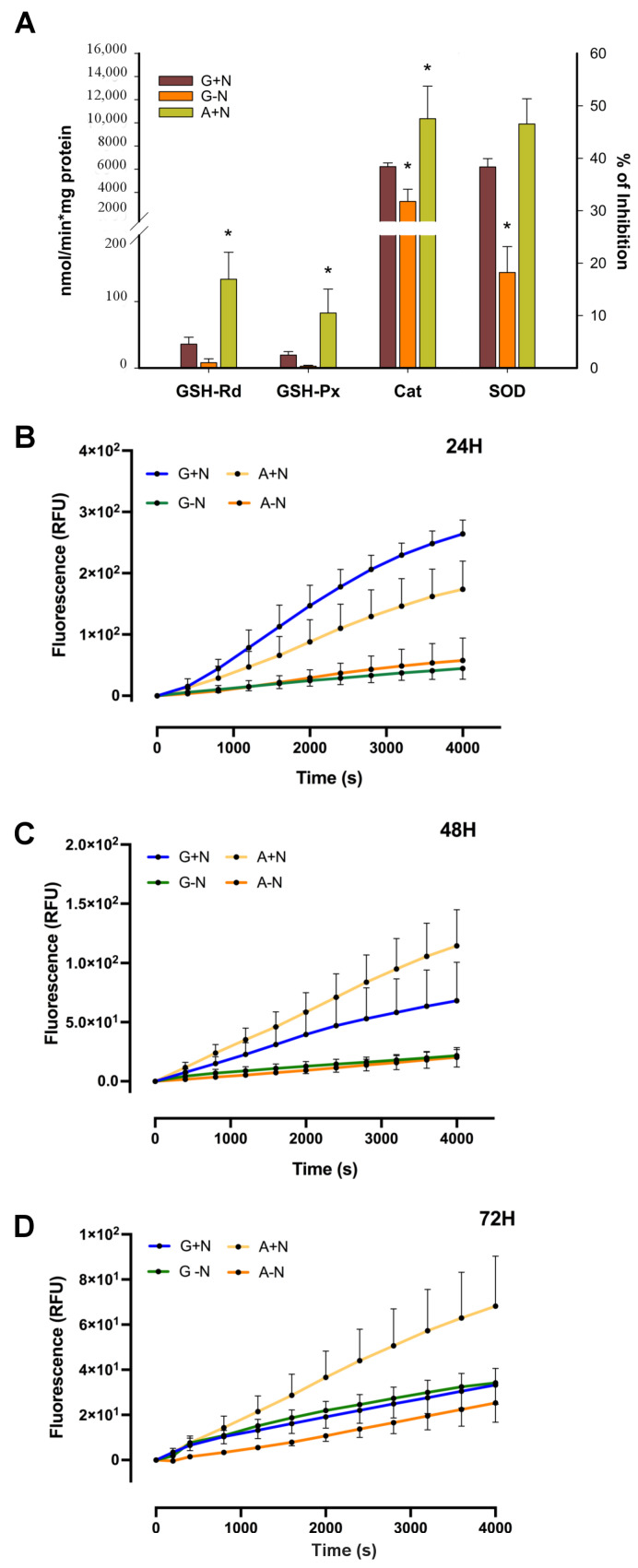
Activities of the antioxidant enzymes and production of hydrogen peroxide. (**A**) Activities of antioxidant enzymes from cells grown for 24 h in different media. (**B**–**D**) Production of hydrogen peroxide by cells harvested at 24 h, 48 h, and 72 h of growth. Abbreviation: GSH-Rd, glutathione reductase; GSH-Px, glutathione peroxidase; Cat, catalase-peroxidase; SOD, superoxide dismutase. SOD, was reported as the % inhibition of NBT reduction. Minimal media containing glucose and nitrogen (G+N), glucose without nitrogen (G-N), minimal media with acetate plus nitrogen (A+N), and acetate without nitrogen (A-N). Data are shown as the mean and standard deviation of 4 independent experiments and their respective standard deviation. * Indicates a significant difference at *p* < 0.05, comparing minimal media with glucose plus nitrogen with the other conditions (ANOVA).

It has been reported that *Saccharomyces cerevisiae* and *U. maydis* cells growing in acetate increased their production of ROS, a condition that finally led to cell death [6,19,39]. Interestingly, in *U. maydis* cultured in the presence of acetate, there was no increase in the expression of genes involved in the antioxidant response, although the enzyme activities were not examined that work [6]. Thus, we analyzed the activities of Cat, SOD, GSH-Rd, and GSH-Px in extracts obtained from cells incubated in the different conditions. Figure 6A shows that all the enzymes were more active in A+N than in G+N or G-N. In the condition A+N, Cat showed an activity of 10,400 ± 2800 nmol min^−1^ mg protein^−1^, 3200 ± 1100 in G-N, and 6200 ± 300 in G+N. The GSH-Rd displayed a specific activity of 134 ± 40 in A+N, three times higher than that in G+N (36 ± 10) or 16 times higher than in G-N (8 ± 6) (Figure 6A). Similarly, in A+N the specific activity of GSH-Px was 83 ± 36, compared to 19 ± 5 in G+N or 2.8 ± 1.4 in G-N (Figure 6A). Another point that stands out is the higher capacity of Cat with respect to the activities of the enzymes of the glutathione system. Because the results indicate that *U. maydis* cells grown on acetate increased the activity of antioxidant enzymes, the next step was to determine the production of H_2_O_2_ by cells (Figure 6B). Production of H_2_O_2_ was assayed with Amplex red kit. At 24 h, cells growing in G+N had the highest production of H_2_O_2_, while at 48 h and 72 h, cells cultured in A+N produced more H_2_O_2_. The results suggest that the growth of *U. maydis* in the presence of acetate induces oxidative stress and that the production of H_2_O_2_ is related to vegetative growth.

## 4. Discussion

### 4.1. Growth and Cell Viability on Acetate as a Carbon Source

The transport of acetic acid across the cell plasma membrane can be achieved by simple diffusion [40] or mediated by four transporters, the two electroneutral acetate/H^+^ symporters Jen1 and Ady2 [41,42], the monocarboxylate carrier, or the aquaglyceroporin channel Fps1 [40,43]. The inhibition of cell growth by acetic acid in many organisms can be explained by at least two factors, the uncoupling effect of weak acids and the acidification of cell cytoplasm due to the dissociation of acetic acid [42,44]. In agreement with this proposal, it has been shown that growth inhibition by acetic acid depends on the external pH, the lower the pH of the medium the greater the inhibition of growth [45]. In fact, inhibition of cell growth by acetic acid has been reported in many fungal species, such as *S. cerevisiae* [42,44,46]. Interestingly, the growth of *U. maydis* cells in A+N was good. At 72 h of growth, cells reached a biomass of 1.6 gDW/L from an initial inoculum of 0.04 gDW/L. Compared to other carbon sources, acetate was an acceptable substrate. *U. maydis* cells grew in acetate with a duplication time of 8.8 h, a value that compares favorably with the duplication times of 3.1 h in G+N, 4.9 h in a minimal medium with ethanol, and 20 h with glycerol or lactate [9]. The simplest explanation is that the alkalinization of the external medium by *U. maydis* cells might avoid the acidification of the intracellular space, allowing the cells to grow and maintain cell viability in acetate as the carbon source. Consistent with our results, other yeast species such as *Hanseniaspora valbyensis*, *Debaryomyces hansenii*, *Rhodotorula glutinis*, *Candida utilis* [47], *S. cerevisiae* [48], and yeast-like cells of *U. maydis* [6] can grow on acetate as the only carbon source. In contrast to our results, it has been reported that after 72 h of growth in acetate, there was severe oxidative stress in *U. maydis,* leading to cell death [6]. We suggest that the decrease in viability reported by Kretschmer (2018) is because the cells are in the stationary phase [6]. In agreement with Kretschmer’s results, there is a decrease in viability when *S. cerevisiae* cells growing in ethanol and acetate entered the stationary phase [39]. However, in our experimental conditions, cells did not reach the stationary phase because the medium still contains acetate. This can be corroborated easily with the value of the biomass yield. Assuming similar biomass yields for two respiratory microorganisms, *Candida utilis* (0.39 g/g acetate) [49] and *U. maydis*, it can be verified that at 72 h the culture medium contains 59% (5.9 g/L) of the initial concentration of acetate. Since the drop in viability depends on the stay of cells in the stationary phase, it makes sense that in our experimental conditions, cells were viable.

An indirect indication of oxidative stress in cells cultured in glucose or acetate is the production of H_2_O_2_. The efflux of H_2_O_2_ into the extracellular medium was higher in cells growing in G+N than in A+N at 24 h of culture, but after that, the production of H_2_O_2_ was higher in cells growing in A+N. Production of H_2_O_2_ probably is associated with cell growth, as indicated more clearly by the condition G+N, because when glucose was consumed and cells stopped growing at 48 h and 72 h, the production of H_2_O_2_ decreased. In the A+N medium, the production of H_2_O_2_ by *U. maydis* cells decreased with the culture time, and it can be hypothesized that this is a consequence of cells decreasing their growth rate and approaching the stationary phase.

### 4.2. Acetate and the Synthesis of TAGs and Glycogen

Our initial working hypothesis was that the production of TAGs by cells could increase in A+N because of the direct assimilation of this molecule into acetyl-CoA, a precursor of fatty acids. This argument was also based on the observation that the lipogenic yeast *Yarrowia lipolytica* can assimilate acetate, convert it to acetyl-CoA and further direct the carbon atoms to fatty acid synthesis [38]. We previously showed that *U. maydis* accumulated a high amount of neutral lipids using glucose as a carbon source and in the absence of a nitrogen source. In contrast with this condition, with acetate there was no accumulation of TAGs in LD in the presence of nitrogen, but without a nitrogen source, there was an increase in LD. In the absence of a nitrogen source, *U. maydis* growth was minimal, but in the presence of nitrate, it seems that cells optimized the use of acetate to grow and build biomass, discarding any large increase in LD synthesis. A similar behavior was observed in cells growing in G+N. Therefore, it appears that when cells are growing with glucose or acetate as carbon sources, the accumulation of LD is limited. However, in the absence of a nitrogen source, a condition that inhibits growth, the accumulation of TAGs and LD occurs regardless of the carbon source. Although the activity of FAS in A+N and G-N was smaller than in G+N, this activity was enough to support growth under the A+N condition and LD synthesis in G-N.

**Figure 7:**
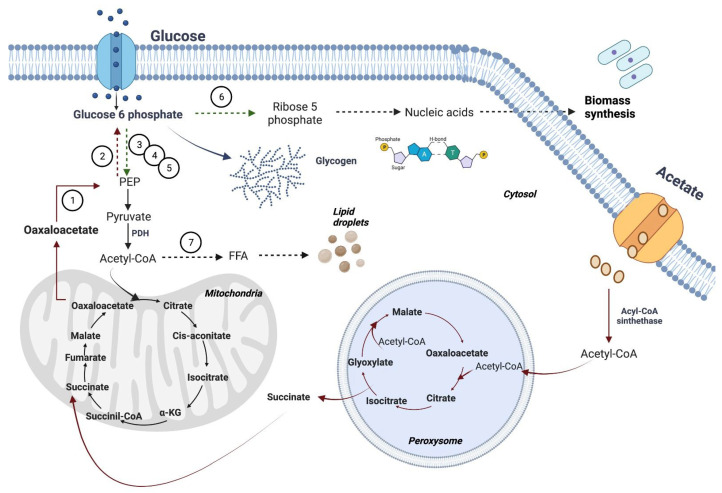
Metabolic pathways of acetate and glucose metabolism in *U. maydis*. The metabolism of acetate is indicated in red arrows (glyoxylate cycle and gluconeogenesis), the glucose metabolism is shown in blue (glycolysis), and the phosphate pentose pathway is shown in green. The numbers inside the circles indicates the enzymes involved in the different steps along the pathways. The abbreviations corresponds to (1) PEPCK, (2) FBPase, (3) HK, (4) PFK-1, (5) PK, (6) G6PDH, (7) FAS. Figure was created with BioRender.com. Agreement number: PG25FVGYFP.

In addition to the production of TAGs under the A+N condition, *U. maydis* also synthesized glycogen as a strategy to store the carbon atoms of acetate (Figure 7). The amount of glycogen in cells at 48 h cannot be explained by the dilution of the original glycogen contained in the inoculum. If this were the case, the amount of glycogen would be 0.44 µg glucose/mgDW instead of the 130–150 µg glucose/mgDW observed experimentally. Therefore, there is a net synthesis of glycogen by *U. maydis* cells in the A+N condition.

### 4.3. Acetate and Futile Cycles

Two points can be extracted from the data regarding enzyme activities. First, the activities of the glycolytic enzymes—except for the PFK1—and the activity of the G6PDH are much higher than the activities of gluconeogenic enzymes, at least an order of magnitude. This implies a greater capacity of glycolysis and the pentose phosphate pathway than that of gluconeogenesis. Given the low specific activity of the PFK1, it is reasonable to assume that the entry route of glucose to the lower part of glycolysis is through the pentose phosphate pathway, as occurs in *Y. lipolytica* [50]. Second, the activities of all the enzymes assayed in G+N are higher than those in G-N. The smaller activities in the G-N condition probably are linked to the fact that the culture of *U. maydis* cells in glucose minus nitrate is associated with the activation of autophagy [16]. As expected, the gluconeogenic enzymes PEPCK, PC, and FBPase were expressed in cells growing on acetate. These enzymes are essential for the assimilation of carbon atoms from acetate into biomass (Figure 7). PEPCK was found only in cells growing on acetate, but the activities of the other two gluconeogenic enzymes, PC and FBPase, were also present in cells cultured in G+N. The presence of PC in all conditions makes sense because, in addition to its gluconeogenic role, PC is important to replenish the pool of oxaloacetate for the functioning of the Krebs cycle. However, the presence of the pair FBPase-PFK1 in cells grown in G+N and A+N, and the pair PEPCK-PK in cells cultured in A+N raises the possibility of futile cycles and the need for regulation of enzyme activities to avoid energy losses (Figure 7).

## 5. Conclusions

Four conclusions can be drawn from our results: (1) In a culture medium containing nitrate, and regardless of the presence of acetate or glucose as a carbon source, the synthesis of lipids is closely associated with the growth of *U. maydis.* However, when the nitrogen source is lacking in the culture media, cells stop growing and the excess of carbons inside the cell are directed towards the synthesis of triacylglycerols which are stored in LD. (2) The increase in the activity of the antioxidant enzymes in cells growing in the A+N medium suggests a higher production of ROS with respect to those cultured in G+N. (3) The production of H_2_O_2_ by *U. maydis*, either in the presence of acetate or glucose in the culture media, was associated with cells that were actively growing. (4) As long as the cells are actively growing in the presence of acetate, they remain viable. However, when cells consumed the acetate in the culture medium and jumped into the stationary phase, viability drops with time.

## Figures and Tables

**Table 1:** Summary of the enzyme activities determined in each condition.

Enzyme	Growth Conditions/Metabolic Pathway		
Gluconeogenesis (nmol min^−1^ mg protein^−1^)			
	G+N	G-N	A+N
HK	122 ± 31	21 ± 3.5	210 ± 41
PFK1	9.8 ± 4	3.8 ± 1.0	7.9 ± 1.16
PK	246 ± 64	116 ± 42	59 ± 24
Pentose phosphate pathway (nmol min^−1^ mg protein^−1^)			
	G+N	G-N	A+N
G6PDH	210 ± 51	47 ± 12	278 ± 76
Gluconeogenesis enzymes (nmol min^−1^ mg protein^−1^)			
	G+N	G-N	A+N
PEPCK	N.D	N.D	36 ± 18
FBPase	8.4 ± 3.4	N.D	20 ± 8.6
Lipid synthesis (nmol min^−1^ mg protein^−1^)			
	G+N	G-N	A+N
FAS	35 ± 8	5.5 ± 2	5.5 ± 2
Antioxidant enzymes (nmol min^−1^ mg protein^−1^)			
	G+N	G-N	A+N
GSH-Px	19 ± 5	2.8 ± 1.4	83 ± 36
GSH-Rd	36 ± 10	8 ± 6	134 ± 40
Cat	6200 ± 300	3200 ± 1100	10,400 ± 2800
SOD	38 ± 1.6	18 ± 5	46.5 ± 4.8

HK, hexokinase. PFK1, phosphofructokinase. PK, pyruvate kinase. G6PDH, glucose-6-phosphate dehydrogenase. PEPCK, phosphoenol pyruvate carboxykinase. FBPase, fructose 1,6 bisphosphatase. FAS, fatty acid synthase. GSH-Px, glutathione peroxidase. GSH-Rd, glutathione reductase. Cat, catalase. SOD, superoxide dismutase. N.D. Not detected. SOD was determined as the % of inhibition of NBT reduction. Data are shown as the mean of 4 independent experiments and their respective standard deviation.

## Data Availability

Not applicable.
